# Atomic Force Microscopy and Scanning Ion-Conductance Microscopy for Investigation of Biomechanical Characteristics of Neutrophils

**DOI:** 10.3390/cells13211757

**Published:** 2024-10-23

**Authors:** Mikhail Shvedov, Ekaterina Sherstyukova, Snezhanna Kandrashina, Vladimir Inozemtsev, Viktoria Sergunova

**Affiliations:** 1 Federal Research and Clinical Center of Intensive Care Medicine and Rehabilitology, V.A. Negovsky Research Institute of General Reanimatology, 107031 Moscow, Russia; kmanchenko@yandex.ru (E.S.); snezhanna.lyapunova@yandex.ru (S.K.); va.inozemcev@physics.msu.ru (V.I.); 2 Koltzov Institute of Development Biology of Russia Academy of Science, 119334 Moscow, Russia

**Keywords:** AFM, SICM, neutrophils, NETosis, elastic modulus, ROS

## Abstract

Scanning probe microscopy (SPM) is a versatile tool for studying a wide range of materials. It is well suited for investigating living matter, for example, in single-cell neutrophil studies. SPM has been extensively utilized to analyze cell physical properties, providing detailed insights into their structural and functional characteristics at the nanoscale. Its long-standing application in this field highlights its essential role in cell biology and immunology research, significantly contributing to understanding cellular mechanics and interactions. In this review, we discuss the application of SPM techniques, specifically atomic force microscopy (AFM) and scanning ion-conductance microscopy (SICM), to study the fundamental functions of neutrophils. In addition, recent advances in the application of SPM in single-cell immunology are discussed. The application of these techniques allows for obtaining data on the morphology, topography, and mechanical and electrochemical properties of neutrophils with high accuracy.

## 1. Introduction

### 1.1. Neutrophils: Functions and Mechanisms

#### 1.1.1. General Characteristics of Neutrophils

**Figure 1:**
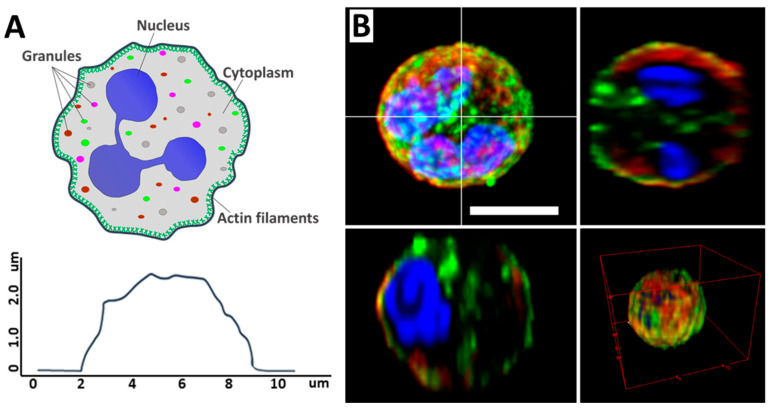
(**A**) Schematic illustration of a neutrophil, its main components (upper image), and cell profile (lower image). Adapted with permission from [5]. Neubert et al., 2018. (**B**) CLSM 2D and 3D images of neutrophil and its profiles. Scale bar 5 μm. The images were acquired by Zeiss LSM880 (Carl Zeiss, Jena, Germany). Image processing, including conversion of imaged z-stacks into maximum intensity projections (MIP), was performed in ImageJ software version 1.53q. Adapted with permission from [6]. Inozemtsev et al., 2023.

Neutrophils are the largest group of myeloid leukocytes, accounting for between 50% and 70% of human white blood cells, and are essential in innate immune defense [1,2]. The main components of the neutrophil are the segmented nucleus, the cytoskeleton, and the cytoplasm, which contains various granules. The neutrophil itself is a spherical cell (Figure 1A) [3]. Neutrophils are formed in the bone marrow from stem cells and have a distinctive segmented nucleus consisting of two to five lobes connected by thin lines of chromatin (Figure 1B) [4].

#### 1.1.2. Neutrophils’ Function in the Immune System

As neutrophils are phagocytic, they possess various mechanisms of pathogen destruction, including phagocytosis, degranulation, the release of reactive oxygen species (ROS), and the formation of neutrophil extracellular traps (NETs) [7]. The process of phagocytosis involves the neutrophil capture of the pathogen, the envelopment of the pathogen by the phagosome, and further destruction [8]. During degranulation, antimicrobial substances and ferments contained in neutrophil granules are released, damaging pathogens’ membranes and destroying them [9,10]. In addition, neutrophils can generate ROS, such as superoxide anion, hydrogen peroxide, and hydroxyl radicals, which have strong antimicrobial properties and contribute to destroying pathogens [11]. Yet another method of pathogen control is the release of NETs, which consist of DNA and associated antimicrobial proteins [12].

### 1.2. Neutrophil Extracellular Traps (NETs)

#### 1.2.1. Mechanisms of NET Formation

There are two mechanisms of NET formation: classical (suicidal) and vital [13]. Suicidal NETosis involves the participation of NADPH-oxidase and ends with neutrophil death, as described by Zychlinsky A. [14]. Agonists of classical NETosis include many microorganisms and pharmacological activators, including phorbol-12-myristate-13-acetate (PMA) [11,15,16,17]. The process starts with the activation of neutrophils by various stimuli such as bacterial components, cytokines, or other pathogens [18,19]. Then, the decondensation of chromatin from neutrophil nuclei occurs, which is associated with the citrullination of the enzyme histones by peptidyl-arginine-diminase 4 (PAD4) [20,21]. After that, the nuclear envelope breaks down, chromatin mixes with cytoplasmic components, and granules bearing antimicrobial proteins such as myeloperoxidase and neutrophil elastase merge with decondensed chromatin [22,23]. The chromatin and granules are then released outward through the disrupted cell membrane, forming NETs, followed by neutrophil death [24,25]. Vital NETosis allows the neutrophil to remain alive after the formation of NETs, and this process is much more rapid. NET formation by this mechanism involves neutrophil activation by components similar to the suicidal mechanism, followed by the release of chromatin and antimicrobial proteins through vesicles, resulting in NET formation while the neutrophil cell membrane remains intact [26]. This mechanism keeps the neutrophil alive and performs its functions after NETs are released.

#### 1.2.2. Biological Significance of NETosis

NETs have been discovered in many organs and tissues [27], and their high concentration is observed in the focus of infections. It is assumed that NETs contribute to slowing down the spread of pathogens due to their net structure [28]. If NET formation is insufficient, it is more complicated for the organism to endure inflammatory processes, as shown in mice affected by necrotizing fasciitis [29].

The mechanism of NET formation depends on the formation of ROS. The article [30] described the dependency of ROS production by neutrophils in chronic granulomatous disease (CGD) patients. Patients with CGD have an impaired NADPH oxidase enzyme complex, which is responsible for the production of ROS, which in turn are an essential component in the formation of NETs. In this disease, neutrophils are unable to form NETs. ROS levels were measured using fluorescence microscopy after the treatment of neutrophils with inhibitors. The study showed a significant formation of NETs after the treatment of neutrophils by CGD patients with PPARγ agonists despite the defect of NADPH oxidase. This indicates that NET formation may occur independently of NADPH oxidase via mitochondrial ROS, enhanced after treatment with PPARγ agonists. However, the excess NET formation also negatively affects the body by causing inflammatory and autoimmune pathologies as well as blood vessel blockages.

Since epithelial barriers such as oral, ocular, and skin mucosa are constantly under attack by microorganisms, the regulation of NET formation must be strictly controlled to avoid inducing inflammation in these tissues [31]. NETs play an essential role in the pathogenesis of platelets of various origins [32]. NETs contribute to the retention of small clots, and their involvement is also determined by their interaction with endothelium and platelets [33]. NETs also take part in the defense of the respiratory tract against infections by increasing mucus viscosity and killing pathogens. NETosis may contribute to the complications of infectious lung diseases such as acute respiratory distress syndrome, chronic obstructive pulmonary disease [34], and bronchial asthma [35]. NET formation occurs in autoimmune diseases, during which NET components such as double-helical DNA, granule proteins, and histones can induce antibody production, thereby promoting autoimmune diseases [36,37,38]. NET formation has been observed in other immune cells, such as eosinophils and mast cells [39], as well as in basophils [40], monocytes [41], and macrophages [42].

Various microscopy methods are used to study the structure and functional properties of cells, such as fluorescence microscopy [43], optical super-resolution methods [44], electron microscopy [45], flow cytometry [46,47,48], atomic force microscopy (AFM) [49], and scanning ion-conductance microscopy (SICM) [50].

Studying the structure of neutrophils and their properties, such as elastic modulus, core swelling, and membrane parameters, allows us to deepen the already existing data on this topic. Various methods are currently used to study the properties of neutrophils, but AFM and SICM allow us to examine the nanostructural, mechanical, and physicochemical properties of cells.

### 1.3. Scanning Probe Microscopy Methods for Investigation of Neutrophils

AFM and SICM offer a wide range of possibilities for biological studies, each with its own advantages and limitations. However, AFM and SICM, as well as other microscopy techniques providing nanometer resolution, are difficult to use and require a researcher’s skills. However, they allow us to estimate cellular parameters that cannot be obtained by classical electron and fluorescence microscopy.

#### 1.3.1. AFM

**Figure 2:**
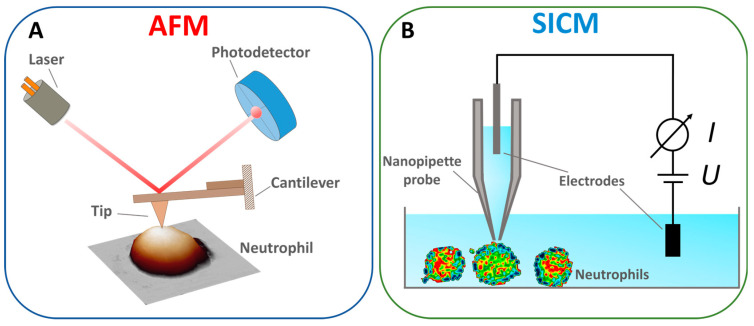
Schematized principle of operation of (**A**) AFM. Adapted with permission from [56]. Tilinova et al., 2024. (**B**) SICM. Adapted with permission from [57]. Happel et al., 2012.

The AFM principle is as follows: the AFM probe, a flexible cantilever with a sharp tip at the end, is brought into interaction with the sample surface (Figure 2A). In the static mode (contact method), the interaction force between the tip and the sample surface is linearly proportional to the vertical bending of the cantilever and its stiffness. The traditional scheme measures the bending of the cantilever by an optical tracking system in which the light source is focused on the surface of the cantilever, and the reflected beam is captured by a sectioned photodetector [51]. The position of the spot on the photodetector is linearly proportional to the cantilever bending and the interaction force between the AFM probe and the sample surface. Linearity is reached by choosing a cantilever force constant. Most AFM methods are based on maintaining a constant force. Along with the contact mode, the resonance (semi-contact) and “jumping” modes are the most common [52,53]. The AFM method is also used to study the mechanical properties of cells, such as the elastic modulus, adhesion forces, and the elastic modulus of components such as the cytoskeleton [54,55].

In addition to the possibility of mapping surface properties with high spatial resolution [58], the great advantage of AFM is the vast possibilities of controlling the measurement conditions: temperature, environment, etc. [59,60,61]. Another significant advantage of AFM is the possibility of studying living cells under physiological conditions. Such a study is possible by using special imaging techniques such as QI or peak force tapping imaging [59,62]. It is also known that modified high-speed atomic force microscopy (HS-AFM) can be used for this purpose. Such a study caused a number of difficulties, including the collision of a cell with a probe, but the improvement of an extremely long (~3 μm) AFM tip attached to a cantilever enables the reduction of severe damage to soft cells [63]. However, little information currently exists on the use of this technique to study live neutrophils. Also, the method has some limitations, including a relatively slow measurement speed [64,65], but even this limitation can be eliminated using HS-AFM [66,67].

#### 1.3.2. SICM

The SICM method uses a thin electrolytic probe to measure ionic conductivity near the sample surface. The system’s core component is a nanopipette probe filled with electrolytes (Figure 2B), whose tip is brought to a short distance from the surface. Samples are scanned by changing the distance between the probe and the sample, measured by the drop in ionic current [68]. In this way, the surface’s morphological, chemical, and electrical properties are mapped with high resolution [69]. This method can also be used for the point injection of drugs [70]. The advantages of the SICM method include non-invasiveness [71], a high resolution for scanning living objects [72], and the ability to measure ionic conductivity [73]. The drawbacks include the complexity of the design, which requires precise tuning of the system to maintain a constant ionic current, slow scanning speed, and limitations on the type of sample; the method is less effective for solid and non-conductive samples, where ionic conductivity does not play a significant role [74].

One of the key advantages of both atomic force microscopy (AFM) and scanning ion-conductance microscopy (SICM) is their ability to study the morphology and determine the nanoscale properties of living biological systems without the need for sample preparation or fluorescent labeling while achieving a resolution that surpasses the diffraction limits typical of optical and fluorescence systems.

## 2. Application of SPM for the Study of Neutrophils and NETs

### 2.1. Morphology by AFM and SICM

AFM and SICM provide detailed information on the structure of morphological changes on the neutrophil surface, which are challenging to study using traditional fluorescence microscopy methods.

**Figure 3:**
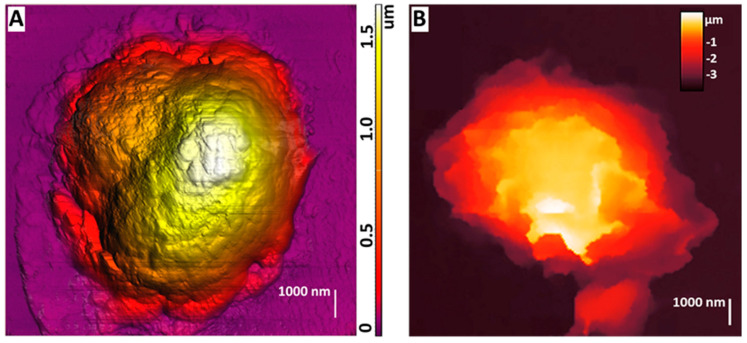
(**A**) AFM image of dry non-activated neutrophils. The image was acquired by NTEGRA Prima AFM (NT−MDT SI, Moscow, Russia), NSG01 cantilever (NT−MDT SI, Moscow, Russia) with a tip radius of 10 nm. The image was processed using Image Analysis P9 software, version 3.5.0.20601 (NT−MDT SI, Moscow, Russia). Adapted with permission from [75]. Sergunova et al., 2023. (**B**) SICM image of a live non-activated neutrophil. The image was acquired by MultiClamp 700 B amplifier (Molecular Devices, Wokingham, UK) using laser-pulled nanopipettes with a 25–40 nm radius. The image was processed using FemtoScan Online software, Version 2.3.239 (5.2) (Advanced Technologies Center, Moscow, Russia, www.nanoscopy.ru (accessed on 21 October 2024)). Adapted with permission from [76]. Pleskova et al., 2020.

AFM could be used to study the morphology of neutrophils during their activation, which was shown at [75] (Figure 3A). By obtaining detailed images of morphological changes in neutrophils, a sequence of stages was outlined: cell expansion, the formation of cell fragments, the fusion of nuclear segments, membrane disruption, NET release, and final cell decay.

The process begins with membrane degradation, after which granular fragments become visible within the intercellular space. Further, nuclear material with cytoplasmic content flows out of the cell in the form of net-like structures consisting of granules, disrupting the cell’s integrity and its membrane. In the case of exposure to A23187 for 120 min, there was a structure change of neutrophils: nuclei lost segmentation and occupied the whole available volume of the cell. In the case when PMA was used as an activator, the nuclei lost segmentation in 60 min, and in 120 min, the cell membranes started to suffer partial destruction. This study showed that their surface becomes smoother and less rough during the activation of neutrophils.

SICM can be used to obtain images of living neutrophils, which was first shown in [76]. The purpose of the article was to study the effect of *S. aureus* on neutrophils and endotheliocytes. This work visualized stiffness maps and neutrophil topography using SICM (Figure 3B). Upon exposure to bacterial agents such as *S. aureus*, neutrophils’ morphology, rigidity, and adhesion were changed. Studies have shown that under the influence of *S. aureus*, the membrane–cytoskeletal complex of neutrophils becomes less rigid, which is reflected by a decrease in their adhesion. These changes are probably related to changes in receptors’ expression and the membrane’s physicochemical properties. For example, adding the *S. aureus* strain to the endotheliocyte–neutrophil system results in a significant decrease in adhesion, indicating a weakening of intercellular interactions.

A visualization of the neutrophil structure using SICM was also shown in [77], in which a comparison of AFM and SICM methods was performed. The simultaneous use of the two methods allowed us to point out the differences in the data obtained. AFM images showed that neutrophils are flat cells with a height of no more than 2 μm in the lobe area and with a large visible cytoplasm area. Since the AFM method uses non-living cells, this may be due to the dehydration of the cells during fixation and their adhesive properties. When visualized by the SICM method, live neutrophils had a distinctive morphology from AFM: most cells were up to 4.5 μm high and did not have a large cytoplasmic area. This may indicate reduced adhesion to the substrate. The study showed that living neutrophils have a variable morphology depending on their physiological state and experimental conditions. The study also demonstrated that a given cell transformed from a spherical cell into a hemispherical cell with apathetic bodies.

### 2.2. Visualization of NET Structure

**Figure 4:**
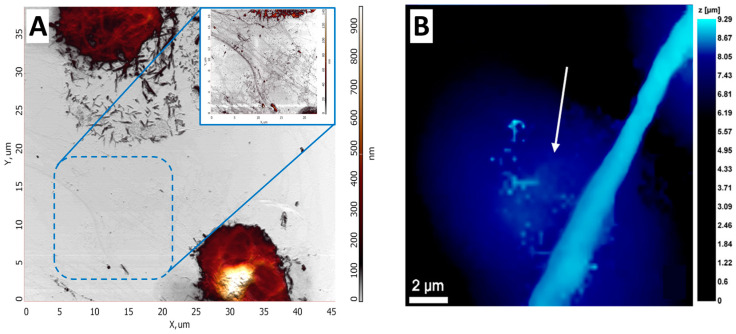
AFM and SICM NETs images. (**A**) AFM image of NETs upon exposure to the activator. Adapted with permission from [75]. Sergunova et al., 2023. (**B**) NET structure obtained by SICM. White arrows indicate NET-like structures. Adapted with permission from [80]. Pleskova et al., 2023.

The authors first visualized NETs using AFM in [78]. They showed that NETs are complex net-like structures composed of filaments organized into branching threads that form a porous network with an average size of about 0.03 µm^2^. The pore size is comparable to the size of small pathogens, which may confirm that NETs function as mechanical “sieves” with elastic properties that are also capable of trapping pathogens. In addition, [75] showed the process of neutrophil transformation under the influence of A23187 as an activator (Figure 4A).

The main objectives of the study [79] were to develop a protocol for NET visualization by AFM and create various techniques for data analysis. The authors obtained images of NETs that revealed a reticular structure with different mechanical characteristics. The data obtained in the study suggest the morphological variability of neutrophils due to structural variations, i.e., the shape and morphology of NETs can significantly differ between systems and within a single structure.

The SICM method was used to visualize changes in the neutrophil structure during transendothelial migration [80]. NET-like structures were detected and visualized using SICM (Figure 4B).

### 2.3. NETosis at Different Time Stages

Applying the AFM method makes it possible to show the NETosis process at different stages and in different periods. In the study [5], the NET formation was divided into three main stages, during which morphological changes occurred in the cells. This work’s results agree with similar studies reported in the literature [6,81]. In addition, NETosis was examined in real time at the single-cell level using AFM, which was shown at [5]. It was found that entropic swelling of chromatin is the primary physical driving force that causes changes in the cell morphology and rupture of both the nuclear and plasma membranes.

The process of NETosis was divided into three separate phases. This phase classification made it possible to distinguish active biological processes from processes occurring without energy input and to determine the point of no return. After cell stimulation, the process proceeded to the first phase, during which the globular structure of the nucleus remained intact, and the corresponding chromatin region was constant. During the second phase, the chromatin expanded for several minutes until it reached the cell membrane as a barrier. The cell membrane burst in the third and last phase, and NETs were released into the extracellular space.

The first phase of NETosis adjusts biochemical modifications, including histone citrullination and lamin phosphorylation [82], which prepares cells for subsequent mechanical changes. The results showed a point of no return, after which active processes, such as enzyme activity, become secondary, and cell behavior is determined by chromatin characteristics. Morphologic changes, as well as the rupture of the nuclear membrane and cell membrane, are caused by entropic swelling of chromatin. At this stage, the pharmaceutical inhibition of NETosis is no longer possible. These data are essential to studying other biological processes, such as cell division or other forms of cell death [83,84].

### 2.4. Neutrophil Elastic Modulus by AFM and SICM Methods

**Figure 5:**
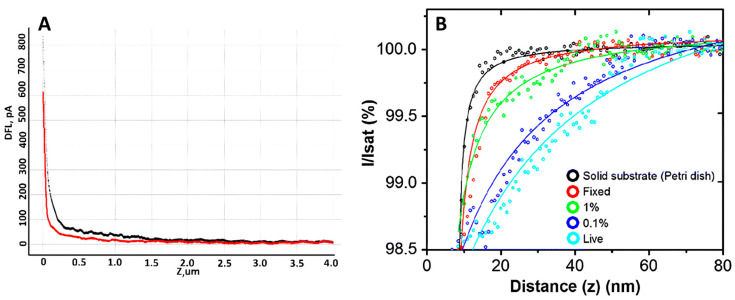
(**A**) An example of force curve, where the black curve is the forward motion and the red curve is the reverse motion. Adapted with permission from [65]. Sergunova et al., 2022. (**B**) Typical ion current–distance curves of the solid substrate (black), a fixed cell (red), a 1% PFA treated cell (green), a 0.1% PFA treated cell (blue) and a live cell (sky blue). Adapted with permission from [91]. Seong-Oh Kim et al., 2017.

Many different techniques can be used to measure cell mechanics; however, AFM and SICM provide high spatial resolution and the ability to quantitatively analyze the mechanical properties of cells. Such properties can change significantly even on the surface of a single cell, so it is crucial to measure several force curves at different points on the cell surface. The number of cells required to obtain reliable statistics is 30 or more [85]. The elastic modulus measurement using AFM has been shown in [86]. The activation of neutrophils was demonstrated by comparing the elastic modulus of control and activated neutrophils. It turned out that after neutrophil activation, Young’s modulus was several times higher compared to control cells. Measuring elastic modulus by AFM requires a specific methodology: measuring the cantilever’s deflection as it moves vertically and observing cell deformation, providing a force-distance curve (Figure 5A). Such curves can be interpreted and analyzed, obtaining quantitative data on elastic modulus [87]. This approach can be used to study single cells or cell layers in culture dishes (in vitro) and tissue samples (ex vivo) [88]. The elastic modulus of neutrophils was measured using the SICM method [76], which allows us to determine the extent to which a cell deforms under mechanical stress. The measurement was performed by analysis of ion current-distance curves (Figure 5B). Curves were obtained by returning a constant ionic current flowing through the nanopipette to approach the cell surface while maintaining a constant tip-to-surface distance approximately equal to the inner radius of the nanopipette [89]. It was shown that the elastic properties of neutrophils differed between the central part of the cell and the periphery. The cell’s deformability was evaluated using Young’s modulus (E). The average E value in the central part of neutrophils was about 765 Pa, while in the cell periphery, it was about 434 Pa. These data indicate a more rigid central region of the cells. Such a non-contact scanning method can provide accurate topography of soft samples with a resolution comparable to AFM. Several other studies have also investigated the elastic modulus of neutrophils. For example, in [90], the influence of structural rearrangements of neutrophil membranes on their ability to deform was considered.

### 2.5. Membrane Parameters

AFM allows us to analyze membrane parameters such as restored adhesion, viscoelastic adhesion, and roughness during neutrophil activation [92]. However, no studies were found in the literature where such neutrophil parameters were measured using SICM. The mapping and analysis of cell surface adhesion properties after fixation provide insights into both the cell surface and the structure of the glycocalyx [83,93,94]. The creation of adhesion maps for neutrophils with different activation times has been demonstrated [56]. When neutrophils are activated, their membrane responds in a specific way. A decrease in local surface adhesion was detected in regions where the membrane had been stretched. The surface adhesion was notably higher in areas where the membrane preserved its folded, original structure.

Fourier spatial transformation is also used to analyze the characteristics of cell membranes, particularly those of neutrophils [90,95]. Using Fourier transformation, the obtained surface profile is decomposed into several simpler components, allowing for the assessment of surface roughness and “wrinkling.” Characteristic membrane structures in neutrophils include unstable, prominent “wrinkles” [90].

### 2.6. Analysis of Nuclear Swelling Using an AFM Probe Beam

Parameters such as the entropic pressure induced by chromatin swelling can be assessed using a cantilever beam. For this purpose, a cantilever beam without a tip was used. After the cell contacts the beam, the height of the z-piezo sensor is kept constant; consequently, cell swelling causes the beam to deviate from its initial position [5].

### 2.7. Intracellular ROS

Assessing neutrophils’ ability to produce ROS is an important task. It is used to study physiological and pathological conditions [96,97,98,99], as neutrophils play a regulatory role as mediators in inflammatory processes [100].

Numerous methods exist for detecting ROS within cells, such as electron paramagnetic resonance [101], fluorescence [102], spectrophotometric methods [103], and chemiluminescence [104]. However, the SICM method is more versatile and convenient for measuring ROS in individual cells. In contrast, the AFM method does not provide the capability to assess ROS. Although fluorescence methods are widely used and have many advantages, they also have some limitations, including measuring signals from a single cell with high temporal resolution. In contrast, SICM allows for measuring intracellular ROS in individual cells [105]. Electrochemical methods are an important approach to the study of ROS. These methods can be employed to assess ROS production in single cells during their activation. Electrochemical sensors enable real-time measurements without the limitations inherent to fluorescent methods.

Recently, platinized nanoelectrodes were developed for the first time, demonstrating their ability to improve the analysis of the production of ROS and reactive nitrogen species (RNS) inside cells. The enhanced sensitivity of these nanoelectrodes enables the detection of deficient intracellular concentrations of ROS, which can be used for cancer research [106]. It has also been demonstrated that using a platinum-carbon microelectrode ammeter allows for the identification and quantification of ROS and RNS [107].

**Figure 6:**
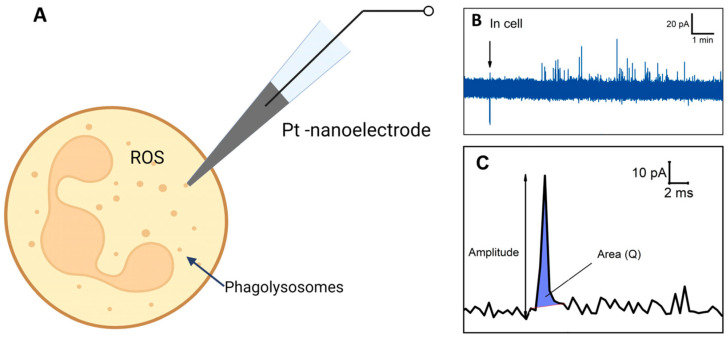
Schematic illustration of experiment. (**A**) Intracellular ROS measurement. (**B**) Chronoamperogram obtained from within the neutrophil. (**C**) Enlarged spike highlighted on the chronoamperogram. Adapted with permission from [108]. Pleskova et al., 2023.

Electrochemical amperometry with high temporal resolution enables the assessment of ROS production in a single neutrophil [108] (Figure 6A). ROS/RNS in phagolysosomes can spill onto the electrochemically active surface and undergo oxidation, resulting in individual amperometric spikes (peaks on chrono-amperometric graphs) (Figure 6B). The spike’s main parameters, namely the area under the spike and amplitude, were measured coulometrically, with the area representing complete ROS molecule oxidation in each phagolysosome. Unstimulated control cells did not exhibit active ROS production. Cells’ response to stimulation varied among neutrophils from the same donor and in cells isolated from different donors. Some cells demonstrated a high level of spike formation, while others did not respond at all. A significant difference in ROS generation was observed when using different stimulators. Upon stimulation with *S. aureus* and *E. coli*, it was observed that the series of peaks begins earlier in the case of *E. coli*. In neutrophils activated by E. coli, the respiratory burst lasted longer but exhibited lower intensity, whereas in neutrophils activated by *S. aureus*, it was shorter and more intense. The charge (spike area) and amplitude were recorded following the release of ROS from the vesicle (Figure 6C). It was also shown that the amount of ROS released from each cell varies and depends on the stimulator: in the case of *S. aureus*, the amount of ROS released is 5.5 times greater compared to *E. coli.* [108].

## 3. Conclusions

Scanning probe microscopy allows us to study neutrophils’ structure and mechanical and electrochemical properties. Only AFM and SICM can describe neutrophil properties such as adhesion, elastic modulus, estimation of ROS within an individual cell with high temporal resolution, and a description of NET formation. The SPM’s ability to record single-cell dynamic processes in real-time mode enables the study of pathophysiological processes with a previously unavailable level of detail. However, some critical disadvantages of SPM methods are also worth mentioning. SPM methods do not provide information about inner structures, such as the location of cytoskeleton proteins. At the same time, SPM, in most cases, cannot provide specific data on cell composition or surface. That is why it is essential to combine SPM methods with other standard methods such as fluorescence or confocal microscopy, Western blot, etc. This will allow for more accurate and qualitative studies and enable more precise correlation analysis of the data obtained through SPM, thereby assisting in their interpretation. Since investigating live samples is an important task in cell biology, specific modifications of AFM and imaging techniques allow such measurements. However, the literature currently lacks sufficient information on the study of live neutrophils using AFM.

In addition, it is worth noting that some technical limitations are inherent to all high-resolution microscopy methods. The limited scanning area makes it difficult to obtain a large number of images, which usually does not allow for large statistics. Probe wear and tear are also limiting factors in the method. SPM is a difficult method for routine measurements due to the abovementioned limitations. However, this method’s capabilities in cell biology allow us to conduct unique experiments to study the stiffness of living cells, obtain data on the cell surface with high spatial resolution, and perform electrochemical measurements in living cells.

## Data Availability

Not applicable.

## References

[1] Garratt L.W. (2021). Current Understanding of the Neutrophil Transcriptome in Health and Disease. Cells.

[2] Ley K., Hoffman H.M., Kubes P., Cassatella M.A., Zychlinsky A., Hedrick C.C., Catz S.D. (2018). Neutrophils: New Insights and Open Questions. Sci. Immunol..

[3] Yu S., Liu J., Yan N. (2022). Endothelial Dysfunction Induced by Extracellular Neutrophil Traps Plays Important Role in the Occurrence and Treatment of Extracellular Neutrophil Traps-Related Disease. Int. J. Mol. Sci..

[4] Vorobjeva N.V. (2020). Neutrophil Extracellular Traps: New Aspects. Mosc. Univ. Biol. Sci. Bull..

[5] Neubert E., Meyer D., Rocca F., Günay G., Kwaczala-Tessmann A., Grandke J., Senger-Sander S., Geisler C., Egner A., Schön M.P. (2018). Chromatin Swelling Drives Neutrophil Extracellular Trap Release. Nat. Commun..

[6] Inozemtsev V., Sergunova V., Vorobjeva N., Kozlova E., Sherstyukova E., Lyapunova S., Chernysh A. (2023). Stages of NETosis Development upon Stimulation of Neutrophils with Activators of Different Types. Int. J. Mol. Sci..

[7] Malech H.L., Deleo F.R., Quinn M.T. (2014). The Role of Neutrophils in the Immune System: An Overview. Methods Mol. Biol..

[8] Harvie E.A., Huttenlocher A. (2015). Neutrophils in Host Defense: New Insights from Zebrafish. J. Leukoc. Biol..

[9] Othman A., Sekheri M., Filep J.G. (2022). Roles of Neutrophil Granule Proteins in Orchestrating Inflammation and Immunity. FEBS J..

[10] Silvestre-Roig C., Hidalgo A., Soehnlein O. (2016). Neutrophil Heterogeneity: Implications for Homeostasis and Pathogenesis. Blood.

[11] Remijsen Q., Berghe T.V., Wirawan E., Asselbergh B., Parthoens E., De Rycke R., Noppen S., Delforge M., Willems J., Vandenabeele P. (2011). Neutrophil Extracellular Trap Cell Death Requires Both Autophagy and Superoxide Generation. Cell Res..

[12] Islam M.M., Takeyama N. (2023). Role of Neutrophil Extracellular Traps in Health and Disease Pathophysiology: Recent Insights and Advances. Int. J. Mol. Sci..

[13] Hidalgo A., Chilvers E.R., Summers C., Koenderman L. (2019). The Neutrophil Life Cycle. Trends Immunol..

[14] Brinkmann V., Reichard U., Goosmann C., Fauler B., Uhlemann Y., Weiss D.S., Weinrauch Y., Zychlinsky A. (2004). Neutrophil Extracellular Traps Kill Bacteria. Science.

[15] Vorobjeva N.V., Pinegin B.V. (2014). Neutrophil Extracellular Traps: Mechanisms of Formation and Role in Health and Disease. Biochemistry.

[16] Ravindran M., Khan M.A., Palaniyar N. (2019). Neutrophil Extracellular Trap Formation: Physiology, Pathology, and Pharmacology. Biomolecules.

[17] Rada B. (2017). Neutrophil Extracellular Traps and Microcrystals. J. Immunol. Res..

[18] Pieterse E., Rother N., Yanginlar C., Hilbrands L.B., van der Vlag J. (2016). Neutrophils Discriminate between Lipopolysaccharides of Different Bacterial Sources and Selectively Release Neutrophil Extracellular Traps. Front. Immunol..

[19] Jorch S.K., Kubes P. (2017). An Emerging Role for Neutrophil Extracellular Traps in Noninfectious Disease. Nat. Med..

[20] Wang Y., Li M., Stadler S., Correll S., Li P., Wang D., Hayama R., Leonelli L., Han H., Grigoryev S.A. (2009). Histone Hypercitrullination Mediates Chromatin Decondensation and Neutrophil Extracellular Trap Formation. J. Cell Biol..

[21] Leppkes M., Maueröder C., Hirth S., Nowecki S., Günther C., Billmeier U., Paulus S., Biermann M., Munoz L.E., Hoffmann M. (2016). Externalized Decondensed Neutrophil Chromatin Occludes Pancreatic Ducts and Drives Pancreatitis. Nat. Commun..

[22] Herrero-Cervera A., Soehnlein O., Kenne E. (2022). Neutrophils in Chronic Inflammatory Diseases. Cell. Mol. Immunol..

[23] Papayannopoulos V., Metzler K.D., Hakkim A., Zychlinsky A. (2010). Neutrophil Elastase and Myeloperoxidase Regulate the Formation of Neutrophil Extracellular Traps. J. Cell Biol..

[24] Vorobjeva N.V., Chernyak B.V. (2020). NETosis: Molecular Mechanisms, Role in Physiology and Pathology. Biochemistry.

[25] Fuchs T.A., Abed U., Goosmann C., Hurwitz R., Schulze I., Wahn V., Weinrauch Y., Brinkmann V., Zychlinsky A. (2007). Novel Cell Death Program Leads to Neutrophil Extracellular Traps. J. Cell Biol..

[26] Desai J., Mulay S.R., Nakazawa D., Anders H.J. (2016). Matters of Life and Death. How Neutrophils Die or Survive along NET Release and Is “NETosis” = Necroptosis?. Cell. Mol. Life Sci..

[27] Yousefi S., Simon D., Stojkov D., Karsonova A., Karaulov A., Simon H.U. (2020). In Vivo Evidence for Extracellular DNA Trap Formation. Cell Death Dis..

[28] Branzk N., Lubojemska A., Hardison S.E., Wang Q., Gutierrez M.G., Brown G.D., Papayannopoulos V. (2014). Neutrophils Sense Microbe Size and Selectively Release Neutrophil Extracellular Traps in Response to Large Pathogens. Nat. Immunol..

[29] Li P., Li M., Lindberg M.R., Kennett M.J., Xiong N., Wang Y. (2010). PAD4 Is Essential for Antibacterial Innate Immunity Mediated by Neutrophil Extracellular Traps. J. Exp. Med..

[30] Hule G.P., Bargir U.A., Kulkarni M., Kambli P., Taur P., Desai M., Madkaikar M.R. (2019). Does Pioglitazone Lead to Neutrophil Extracellular Traps Formation in Chronic Granulomatous Disease Patients?. Front. Immunol..

[31] Yang H., Biermann M.H., Brauner J.M., Liu Y., Zhao Y., Herrmann M. (2016). New Insights into Neutrophil Extracellular Traps: Mechanisms of Formation and Role in Inflammation. Front. Immunol..

[32] Martinod K., Wagner D.D. (2014). Thrombosis: Tangled up in NETs. Blood.

[33] Gupta A.K., Joshi M.B., Philippova M., Erne P., Hasler P., Hahn S., Resink T.J. (2010). Activated Endothelial Cells Induce Neutrophil Extracellular Traps and Are Susceptible to NETosis-Mediated Cell Death. FEBS Lett..

[34] Gajnitdinova V.V., Sharafutdinova L.A., Kamaltdinov I.M., Avdeev S.N. (2014). Investigation of Blood Neutrophil Structural and Functional Characteristics in Patients with Chronic Obstructive Pulmonary Disease and Pulmonary Hypertension Using Atomic Force Microscopy. Russ. Pulmonol..

[35] Caudrillier A., Kessenbrock K., Gilliss B.M., Nguyen J.X., Marques M.B., Monestier M., Toy P., Werb Z., Looney M.R. (2012). Platelets Induce Neutrophil Extracellular Traps in Transfusion-Related Acute Lung Injury. J. Clin. Investig..

[36] Jiménez-Alcázar M., Rangaswamy C., Panda R., Bitterling J., Simsek Y.J., Long A.T., Bilyy R., Krenn V., Renné C., Renné T. (2017). Host DNases Prevent Vascular Occlusion by Neutrophil Extracellular Traps. Science.

[37] Kaplan M.J., Radic M. (2012). Neutrophil Extracellular Traps: Double-Edged Swords of Innate Immunity. J. Immunol..

[38] Kessenbrock K., Krumbholz M., Schönermarck U., Back W., Gross W.L., Werb Z., Gröne H.J., Brinkmann V., Jenne D.E. (2009). Netting Neutrophils in Autoimmune Small-Vessel Vasculitis. Nat. Med..

[39] Von Köckritz-Blickwede M., Goldmann O., Thulin P., Heinemann K., Norrby-Teglund A., Rohde M., Medina E. (2008). Phagocytosis-Independent Antimicrobial Activity of Mast Cells by Means of Extracellular Trap Formation. Blood.

[40] Morshed M., Hlushchuk R., Simon D., Walls A.F., Obata-Ninomiya K., Karasuyama H., Djonov V., Eggel A., Kaufmann T., Simon H.-U. (2014). NADPH Oxidase-Independent Formation of Extracellular DNA Traps by Basophils. J. Immunol..

[41] Granger V., Faille D., Marani V., Noël B., Gallais Y., Szely N., Flament H., Pallardy M., Chollet-Martin S., de Chaisemartin L. (2017). Human Blood Monocytes Are Able to Form Extracellular Traps. J. Leukoc. Biol..

[42] Chow O.A., Von Köckritz-Blickwede M., Bright A.T., Hensler M.E., Zinkernagel A.S., Cogen A.L., Gallo R.L., Monestier M., Wang Y., Glass C.K. (2010). Statins Enhance Formation of Phagocyte Extracellular Traps. Cell Host Microbe.

[43] Schöenfeld L., Appl B., Pagerols-Raluy L., Heuer A., Reinshagen K., Boettcher M. (2023). Immunofluorescence Imaging of Neutrophil Extracellular Traps in Human and Mouse Tissues. J. Vis. Exp..

[44] Stehr A.M., Wang G., Demmler R., Stemmler M.P., Krug J., Tripal P., Schmid B., Geppert C.I., Hartmann A., Muñoz L.E. (2022). Neutrophil Extracellular Traps Drive Epithelial-Mesenchymal Transition of Human Colon Cancer. J. Pathol..

[45] Saha T., Dash C., Jayabalan R., Khiste S., Kulkarni A., Kurmi K., Mondal J., Majumder P.K., Bardia A., Jang H.L. (2022). Intercellular Nanotubes Mediate Mitochondrial Trafficking between Cancer and Immune Cells. Nat. Nanotechnol..

[46] Fan Y., Teng Y., Liu F.T., Ma F., Hsu A.Y., Feng S., Luo H.R. (2023). Neutrophil Lifespan Extension with CLON-G and an In Vitro Spontaneous Death Assay. J. Vis. Exp..

[47] Dumont B.L., Neagoe P.E., Charles E., Villeneuve L., Ninni S., Tardif J.C., Räkel A., White M., Sirois M.G. (2024). Low-Density Neutrophils and Neutrophil Extracellular Traps (NETs) Are New Inflammatory Players in Heart Failure. Can. J. Cardiol..

[48] Liu J., Li Z., Li M., Du W., Baumeister W., Yang J., Guo Q. (2023). Vimentin Regulates Nuclear Segmentation in Neutrophils. Proc. Natl. Acad. Sci. USA.

[49] Qiu Y., Chien C.C., Maroulis B., Bei J., Gaitas A., Gong B. (2022). Extending Applications of AFM to Fluidic AFM in Single Living Cell Studies. J. Cell Physiol..

[50] Dubkov S., Overchenko A., Novikov D., Kolmogorov V., Volkova L., Gorelkin P., Erofeev A., Parkhomenko Y. (2023). Single-Cell Analysis with Silver-Coated Pipette by Combined SERS and SICM. Cells.

[51] Xia F., Youcef-Toumi K. (2022). Review: Advanced Atomic Force Microscopy Modes for Biomedical Research. Biosensors.

[52] Maver U., Velnar T., Gaberšček M., Planinšek O., Finšgar M. (2016). Recent Progressive Use of Atomic Force Microscopy in Biomedical Applications. TrAC Trends Anal. Chem..

[53] Newton R., Müller D.J. (2018). Cells Stiffen for Cytokines. Cell Chem. Biol..

[54] Chattrakun K., Schaefer K.G., Chandler L.S., Marsh B.P., King G.M. (2021). Atomic Force Microscopy Reveals Membrane Protein Activity at the Single Molecule Level. Methods Mol. Biol..

[55] Li M., Dang D., Liu L., Xi N., Wang Y. (2017). Atomic Force Microscopy in Characterizing Cell Mechanics for Biomedical Applications: A Review. IEEE Trans. Nanobioscience.

[56] Tilinova O.M., Inozemtsev V., Sherstyukova E., Kandrashina S., Pisarev M., Grechko A., Vorobjeva N., Sergunova V., Dokukin M.E. (2024). Cell Surface Parameters for Accessing Neutrophil Activation Level with Atomic Force Microscopy. Cells.

[57] Happel P., Thatenhorst D., Dietzel I.D. (2012). Scanning Ion Conductance Microscopy for Studying Biological Samples. Sensors.

[58] Chang K.C., Chiang Y.W., Yang C.H., Liou J.W. (2012). Atomic Force Microscopy in Biology and Biomedicine. Tzu Chi Med. J..

[59] Chopinet L., Formosa C., Rols M.P., Duval R.E., Dague E. (2013). Imaging Living Cells Surface and Quantifying Its Properties at High Resolution Using AFM in QI^TM^ Mode. Micron.

[60] Cheong L.Z., Zhao W., Song S., Shen C. (2019). Lab on a Tip: Applications of Functional Atomic Force Microscopy for the Study of Electrical Properties in Biology. Acta Biomater..

[61] Essmann C.L., Martinez-Martinez D., Pryor R., Leung K.Y., Krishnan K.B., Lui P.P., Greene N.D.E., Brown A.E.X., Pawar V.M., Srinivasan M.A. (2020). Mechanical Properties Measured by Atomic Force Microscopy Define Health Biomarkers in Ageing C. Elegans. Nat. Commun..

[62] Hu J., Chen S., Huang D., Zhang Y., Lü S., Long M. (2020). Global Mapping of Live Cell Mechanical Features Using PeakForce QNM AFM. Biophys. Rep..

[63] Shibata M., Watanabe H., Uchihashi T., Ando T., Yasuda R. (2017). High-Speed Atomic Force Microscopy Imaging of Live Mammalian Cells. Biophys. Physicobiol.

[64] Wu Y., Cai J., Cheng L., Xu Y., Lin Z., Wang C., Chen Y. (2006). Atomic Force Microscope Tracking Observation of Chinese Hamster Ovary Cell Mitosis. Micron.

[65] Sergunova V., Leesment S., Kozlov A., Inozemtsev V., Platitsina P., Lyapunova S., Onufrievich A., Polyakov V., Sherstyukova E. (2022). Investigation of Red Blood Cells by Atomic Force Microscopy. Sensors.

[66] Ando T. (2018). High-Speed Atomic Force Microscopy and Its Future Prospects. Biophys. Rev..

[67] Uchihashi T., Ganser C. (2020). Recent Advances in Bioimaging with High-Speed Atomic Force Microscopy. Biophys. Rev..

[68] Hansma P.K., Drake B., Marti O., Gould S.A.C., Prater C.B. (1989). The Scanning Ion-Conductance Microscope. Science.

[69] Gorelik J., Shevchuk A., Ramalho M., Elliott M., Lei C., Higgins C.F., Lab M.J., Klenerman D., Krauzewicz N., Korchev Y. (2002). Scanning Surface Confocal Microscopy for Simultaneous Topographical and Fluorescence Imaging: Application to Single Virus-like Particle Entry into a Cell. Proc. Natl. Acad. Sci. USA.

[70] Liu B.C., Lu X.Y., Song X., Lei K.Y., Alli A.A., Bao H.F., Eaton D.C., Ma H.P. (2012). Scanning Ion Conductance Microscopy: A Nanotechnology for Biological Studies in Live Cells. Front. Physiol..

[71] Shevchuk A.I., Frolenkov G.I., Sánchez D., James P.S., Freedman N., Lab M.J., Jones R., Klenerman D., Korchev Y.E. (2006). Imaging Proteins in Membranes of Living Cells by High-Resolution Scanning Ion Conductance Microscopy. Angew. Chem. Int. Ed. Engl..

[72] Li C., Johnson N., Ostanin V., Shevchuk A., Ying L., Korchev Y., Klenerman D. (2008). High Resolution Imaging Using Scanning Ion Conductance Microscopy with Improved Distance Feedback Control. Prog. Nat. Sci..

[73] Zhu C., Huang K., Siepser N.P., Baker L.A. (2021). Scanning Ion Conductance Microscopy. Chem. Rev..

[74] Cervera J., Schiedt B., Neumann R., Mafá S., Ramírez P. (2006). Ionic Conduction, Rectification, and Selectivity in Single Conical Nanopores. J. Chem. Phys..

[75] Sergunova V., Inozemtsev V., Vorobjeva N., Kozlova E., Sherstyukova E., Lyapunova S., Chernysh A. (2023). Morphology of Neutrophils during Their Activation and NETosis: Atomic Force Microscopy Study. Cells.

[76] Pleskova S.N., Kriukov R.N., Bobyk S.Z., Boryakov A.V., Gorelkin P.V., Erofeev A.S. (2020). Conditioning Adhesive Contacts between the Neutrophils and the Endotheliocytes by Staphylococcus Aureus. J. Mol. Recognit..

[77] Bezrukov N.A., Pleskova S.N., Bobyk S.Z., Boryakov A.V. (2022). High-Resolution Scanning Ion-Conductance Microscopy for the Study of Blood Cell Morphology and Rigidity. Opera Med. Physiol..

[78] Pires R.H., Felix S.B., Delcea M. (2016). The Architecture of Neutrophil Extracellular Traps Investigated by Atomic Force Microscopy. Nanoscale.

[79] Pires R.H., Delcea M., Felix S.B. (2019). Imaging and Manipulation of Extracellular Traps by Atomic Force Microscopy. Methods Mol. Biol..

[80] Pleskova S.N., Bezrukov N.A., Gorshkova E.N., Bobyk S.Z., Lazarenko E.V. (2023). Exploring the Process of Neutrophil Transendothelial Migration Using Scanning Ion-Conductance Microscopy. Cells.

[81] Kenny E.F., Herzig A., Krüger R., Muth A., Mondal S., Thompson P.R., Brinkmann V., von Bernuth H., Zychlinsky A. (2017). Diverse Stimuli Engage Different Neutrophil Extracellular Trap Pathways. eLife.

[82] Amulic B., Knackstedt S.L., Abu Abed U., Deigendesch N., Harbort C.J., Caffrey B.E., Brinkmann V., Heppner F.L., Hinds P.W., Zychlinsky A. (2017). Cell-Cycle Proteins Control Production of Neutrophil Extracellular Traps. Dev. Cell.

[83] Fisher T.E., Marszalek P.E., Fernandez J.M. (2000). Stretching Single Molecules into Novel Conformations Using the Atomic Force Microscope. Nat. Struct. Biol..

[84] Stewart M.P., Helenius J., Toyoda Y., Ramanathan S.P., Muller D.J., Hyman A.A. (2011). Hydrostatic Pressure and the Actomyosin Cortex Drive Mitotic Cell Rounding. Nature.

[85] Sokolov I., Dokukin M.E., Guz N.V. (2013). Method for Quantitative Measurements of the Elastic Modulus of Biological Cells in AFM Indentation Experiments. Methods.

[86] Roca-Cusachs P., Almendros I., Farré R., Navajas D. (2006). Neutrophil Microrheology Probed by Atomic Force Microscopy. FASEB J..

[87] Cappella B., Dietler G. (1999). Force-Distance Curves by Atomic Force Microscopy. Surf. Sci. Rep..

[88] Guz N., Dokukin M., Kalaparthi V., Sokolov I. (2014). If Cell Mechanics Can Be Described by Elastic Modulus: Study of Different Models and Probes Used in Indentation Experiments. Biophys. J..

[89] Kolmogorov V.S., Erofeev A.S., Barykin E.P., Timoshenko R.V., Lopatukhina E.V., Kozin S.A., Gorbacheva L.R., Salikhov S.V., Klyachko N.L., Mitkevich V.A. (2023). Scanning Ion-Conductance Microscopy for Studying β-Amyloid Aggregate Formation on Living Cell Surfaces. Anal. Chem..

[90] Kozlova E., Sergunova V., Inozemtsev V., Sherstyukova E., Kozlov A., Gudkova O., Chernysh A. (2022). Structural Configuration of Blood Cell Membranes Determines Their Nonlinear Deformation Properties. Biomed. Res. Int..

[91] Kim S.O., Kim J., Okajima T., Cho N.J. (2017). Mechanical Properties of Paraformaldehyde-Treated Individual Cells Investigated by Atomic Force Microscopy and Scanning Ion Conductance Microscopy. Nano Converg..

[92] Sergey A., Viliya G., Lucie S., Ilnur K. (2016). Neutrophils’ Atomic Force Microscopy in COPD with Pulmonary Hypertension (PH). Eur. Respir. J..

[93] Kolesov D., Astakhova A., Galdobina M., Moskovtsev A., Kubatiev A., Sokolovskaya A., Ukrainskiy L., Morozov S. (2023). Scanning Probe Microscopy Techniques for Studying the Cell Glycocalyx. Cells.

[94] Moran H., Cancel L.M., Mayer M.A., Qazi H., Munn L.L., Tarbell J.M. (2019). The Cancer Cell Glycocalyx Proteoglycan Glypican-1 Mediates Interstitial Flow Mechanotransduction to Enhance Cell Migration and Metastasis. Biorheology.

[95] Hartman R.S., Lau K., Chou W., Coates T.D. (1994). The Fundamental Motor of the Human Neutrophil Is Not Random: Evidence for Local Non-Markov Movement in Neutrophils. Biophys. J..

[96] van Gemmeren T., Schuppner R., Grosse G.M., Fering J., Gabriel M.M., Huber R., Worthmann H., Lichtinghagen R., Weissenborn K. (2020). Early Post-Stroke Infections Are Associated with an Impaired Function of Neutrophil Granulocytes. J. Clin. Med..

[97] Barnes T.C., Anderson M.E., Edwards S.W., Moots R.J. (2012). Neutrophil-Derived Reactive Oxygen Species in SSc. Rheumatology.

[98] Umeda T., Takahashi I., Danjo K., Matsuzaka M., Nakaji S. (2011). Changes in Neutrophil Immune Functions under Different Exercise Stresses. Nihon Eiseigaku Zasshi.

[99] Nexar-Quispe Huaman J., Caleiro Seixas A.E. (2019). Adrenergic and Cholinergic Influence on the Production of Reactive Oxygen Species in Human Neutrophils. Rev. Peru. Med. Exp. Salud Publica.

[100] Domerecka W., Homa-Mlak I., Mlak R., Michalak A., Wilińska A., Kowalska-Kępczyńska A., Dreher P., Cichoż-Lach H., Małecka-Massalska T. (2022). Indicator of Inflammation and NETosis—Low-Density Granulocytes as a Biomarker of Autoimmune Hepatitis. J. Clin. Med..

[101] Dikalov S.I., Polienko Y.F., Kirilyuk I. (2018). Electron Paramagnetic Resonance Measurements of Reactive Oxygen Species by Cyclic Hydroxylamine Spin Probes. Antioxid. Redox Signal..

[102] Wojtala A., Bonora M., Malinska D., Pinton P., Duszynski J., Wieckowski M.R. (2014). Methods to Monitor ROS Production by Fluorescence Microscopy and Fluorometry. Methods Enzymol..

[103] Zhang Y., Dai M., Yuan Z. (2018). Methods for the Detection of Reactive Oxygen Species. Anal. Methods.

[104] Yu W., Zhao L. (2021). Chemiluminescence Detection of Reactive Oxygen Species Generation and Potential Environmental Applications. TrAC Trends Anal. Chem..

[105] Tikhonova T.N., Kolmogorov V.S., Timoshenko R.V., Vaneev A.N., Cohen-Gerassi D., Osminkina L.A., Gorelkin P.V., Erofeev A.S., Sysoev N.N., Adler-Abramovich L. (2022). Sensing Cells-Peptide Hydrogel Interaction In Situ via Scanning Ion Conductance Microscopy. Cells.

[106] Vaneev A.N., Gorelkin P.V., Garanina A.S., Lopatukhina H.V., Vodopyanov S.S., Alova A.V., Ryabaya O.O., Akasov R.A., Zhang Y., Novak P. (2020). In Vitro and In Vivo Electrochemical Measurement of Reactive Oxygen Species After Treatment with Anticancer Drugs. Anal. Chem..

[107] Della Valle E., Welle E.J., Chestek C.A., Weiland J.D. (2021). Compositional and Morphological Properties of Platinum-Iridium Electrodeposited on Carbon Fiber Microelectrodes. J. Neural Eng..

[108] Pleskova S.N., Erofeev A.S., Vaneev A.N., Gorelkin P.V., Bobyk S.Z., Kolmogorov V.S., Bezrukov N.A., Lazarenko E.V. (2023). ROS Production by a Single Neutrophil Cell and Neutrophil Population upon Bacterial Stimulation. Biomedicines.

